# Supplementary material for: Directional dipole dice enabled by anisotropic chirality
Source: Proc Natl Acad Sci U S A. 2023 Jun 12;120(25):e2301620120. doi: 10.1073/pnas.2301620120 (PMC10288596; doi:10.1073/pnas.2301620120)
Supplement: Supplementary file 1 — Appendix 01 (PDF) [file pnas.2301620120.sapp.pdf]

Supporting Information for

Directional Dipole Dice Enabled by Anisotropic Chirality

Yuqiong Cheng, Kayode Adedotun Oyesina, Bo Xue, Dangyuan Lei, Alex M. H. Wong and Shubo Wang

Alex M. H. Wong; Shubo Wang.
E-mail: alex.mh.wong@cityu.edu.hk; shubwang@cityu.edu.hk

This PDF file includes:

Supporting text
Figs. S1 to S8
SI References

Supporting Information Text

Eigenmodes of the nanorod. Figure S1 shows the eigenfrequency ω_n of the gold nanorod ($r = 11$ nm) for the first eigenmode $n = 1$ and propagation constant γ_n of the nanorod current for the eigenmodes of orders $n = 1, 2, 3, 4$ under different lengths. The cross symbols denote the analytical results obtained using Eq. (11) (see Materials and Methods), which agree well with the numerical results obtained using COMSOL (denoted by the circle symbols).

Alternative design of the helix as the DDD. The physical mechanism of realizing the directional dipoles in an anisotropic chiral particle is robust, and the phenomena can be demonstrated in different designs of the helix working at different frequencies. In addition to the design in the main text, another design of the helix is shown in Fig. S2*A*, where the pitch is $P = 90$ nm, the outer radius is $R = 40$ nm, and the inner radius is $r = 15$ nm. This helix works at the dipole resonant frequency of 147 THz, and it can give rise to the three types of directional dipole when the incident angle is 15 degrees, as shown in Fig. S2*B*.

Directionality flipping of the DDD. The directionality of the DDD can be flipped by activating the remaining three directional-dipole faces pointing in $-x$, $+y$, and $+z$ directions respectively. Following the settings of the helix-waveguide configurations in Figs. 4-6 (see Results), we reverse the directionality of all types of directional dipoles by tuning the incidence. As seen in Figs. S3*A* and S3*B*, when the incident angle $\theta = 165$ degrees and polarization angle $\varphi = -5$ degrees, the helix can serve as an optimized circular dipole ($p_z/p_x = -1.2\mathrm{i}$) with the directionality of 32 pointing in $-z$ direction, which can be clearly observed in the H_y field of the system in Fig. S3*C*. For the Huygens-dipole face, when $\theta = 175$ degree and $\varphi = -5$ degrees, the helix can realize an optimized Huygens dipole ($p_z/(m_y/c) = 3.6$) with the directionality of 38 pointing in $-x$ direction as shown in Figs. S4*A* and S4*B*. Fig. S4*C* clearly exhibits the unidirectional excitation of the $-x$-propagating guided wave. Figure S5 shows the reversed directionality of Janus-dipole face. When $\theta = -28$ degrees and $\varphi = -68$ degrees, the helix can generate the optimized Janus dipole ($p_x/(m_y/c) = -2.5\mathrm{i}$) and predominantly excites the guided wave when it locates above the waveguide with a directionality of 22. It can also be clearly observed in the electric field distribution (E_x) of the system in Fig. S5*C*.

High-dimensional directional system. To unify all types of directionality in one space and realize the switching among them, we construct a high-dimensional directional system as shown in Fig. S6*A*. Following the schematic in Fig. 1, the helix is surrounded by three sets of waveguide channels with the gap distance $d = 925$ nm, where the transparent parts of the waveguide contain loss. The amplitude ratio of the guided waves in $\pm z$-, $\pm x$-, and $\pm y$-propagating channels corresponds to the directionality of circular dipole, Huygens dipole, and Janus dipole, respectively. The optimization of each dipole and the directional coupling will be influenced by the couplings between the nearby waveguides. By controlling the propagation and polarization directions (i.e., θ and φ) of the incident plane wave, we can achieve complete manipulation of directionality via different directional-dipole faces in orthogonal orientations. In Fig. S6*B*, when $\theta = 8$ degrees and $\varphi = -5$ degrees, we achieve the circular dipole-type directionality with $\mathbf{D}^{\mathrm{e}}_{\mathrm{cir}} = (p_x\hat{\mathbf{e}}_x, p_z\hat{\mathbf{e}}_z)$ in the helix and guide light predominantly propagating in $+z$ direction. The optimized directionality by the dipole $p_z/p_x \approx -0.6 + 2.1\mathrm{i}$ can be clearly observed in the electric field distribution of the system (zx-view) in the inset, which reaches the value of almost 13. Similarly, when $\theta = 20$ degrees and $\varphi = -25$ degrees, we realize the Huygens dipole-type directionality with $\mathbf{D}_{\mathrm{Huy}} = (p_z\hat{\mathbf{e}}_z, m_y\hat{\mathbf{e}}_y)$ in the helix and guide light predominantly propagating in $+x$ direction, as shown in Fig. S6*C*. From the inset, the optimized directionality by the dipole $p_z/(m_y/c) \approx -0.75$, described by the electric field distribution on the xy-plane, reaches about 8. As shown in Fig. S6*D*, when $\theta = -36$ degrees and $\varphi = -67$ degrees, we can achieve the Janus dipole-type directionality with $\mathbf{D}_{\mathrm{Jan}} = (p_x\hat{\mathbf{e}}_x, m_y\hat{\mathbf{e}}_y)$ in the helix and guide light predominantly propagating along $-y$ direction in the lower waveguide. The inset clearly shows the optimized directionality by the dipole $p_x/(m_y/c) \approx -1 - 1.4\mathrm{i}$ via the electric field distribution of the system (yz-view), which reaches the value of nearly 9. An alternative way for the Janus-dipole face to demonstrate the directionality is to truncate the waveguide at an appropriate position of the transparent part. When the reflected wave from the termination constructively interferes with the wave traveling in $+y$ ($-y$) direction in the upper (lower) waveguide, one can also achieve unidirectional propagation of the guided wave in $+y$ or $-y$ direction. Meanwhile, this method can improve the transmission efficiency by harvesting light into one waveguide channel.

Dielectric waveguide to metallic waveguide transition. The dielectric waveguide ($\mu_r = 1, \varepsilon_r = 12$) used in this experiment with cross-sectional dimension of 30 mm $\times$ 15 mm and a total length of 620 mm was fabricated by CNC technology. In opposition to what is obtainable in a metallic waveguide, the fields are not totally confined to the guiding structure in a dielectric waveguide. Transitioning between these two types of waveguides thus presents an interesting challenge. Many transition methods have been proposed over the years (1, 2). In this experiment, we adopt the end tapering method to ensure optimum transition from the dielectric waveguide to the standard WR430 metallic waveguide launchers, which are connected to the vector network analyzer. The two ends of the dielectric waveguide thus have a 60 mm-long taper.

An HFSS simulation setup to confirm the efficient transition is shown in the inset of Fig. S7. We excite Port 1 and measure the received power at Port 2 (S_{21}) as well as the reflected power back into Port 1, comprising the reflection from the metallic waveguide launcher (at Port 1) and the reflection from the end of the dielectric waveguide (at Port 2) back into Port 1 (S_{11}). We also experimentally extract the reflection and transmission parameters when the two ends of the dielectric waveguide are terminated with WR430 metallic waveguide launchers. The WR430 waveguides launchers have a coaxial-to-waveguide adaptor, through which the waveguides are connected to a Vector Network Analyzer (VNA). Port 1 is excited while Port 2 serves as the receiver. The simulated and measured results as presented in Fig. S7 show minimal reflection and good transmission at 2.35 GHz. The results show efficient coupling of the waves from the dielectric waveguide to the metallic waveguides.

The rotated Janus dipole. To enable efficient excitation of the Janus dipole, we employ a slightly different configuration in the microwave experiment, where the Janus dipole is rotated in the xy plane, as shown in Fig. S8*A*. The helix is sandwiched by two waveguides with the gap distance of 75 mm. The incidence is a linearly polarized plane wave in the form of $\mathbf{E}_{\text{inc}} = (-\cos\theta\hat{\mathbf{e}}_x - \sin\theta\hat{\mathbf{e}}_y)E_0 e^{(\mathrm{i}k_0 \sin\theta x - \mathrm{i}k_0 \cos\theta y)}$, where θ is the incident angle between the wavevector $\mathbf{k}$ (on the xy plane) and the $-y$ axis. The optimized Janus dipole can be achieved via tuning the rotation angle ψ of the helix axis with respect to the y axis on the xy plane, i.e., tuning the match between the dipoles (p_x and m_y) and the waveguide mode (E_x and B_y) so that $\left|p_x E_x^* + m_y B_y^*\right| \to 0$, which gives $p_x/(m_y/c) = -cB_y^*/E_x^* = 1.8\mathrm{i}$, at incident angle $\theta = 40$ degrees and rotation angle $\psi = 32$ degrees, as shown in Fig. S8*B*. Figure S8*C* shows that the optimized Janus dipole in the helix predominantly excites the guided wave in the upper waveguide. Figure S8*D* shows the electric field distribution $|E_x|$ in the upper (lower) waveguide, as denoted by the solid (dashed) blue line, which exhibits a high directionality of 20.

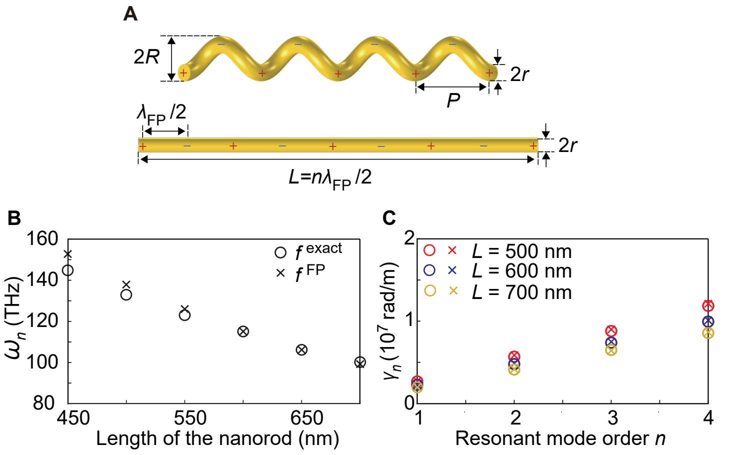

Fig. S1. Eigenmode properties of the nanorod. (*A*) Charge distribution for the Fabry-Perot standing wave of order n in the helix and the nanorod. (*B*) Comparison between analytical results (cross) and numerical results (circle) of the eigen frequency for different lengths of the nanorod. (*C*) Comparison of the propagation constants of the eigenmodes obtained by the analytical method (cross) and numerical method (circle).

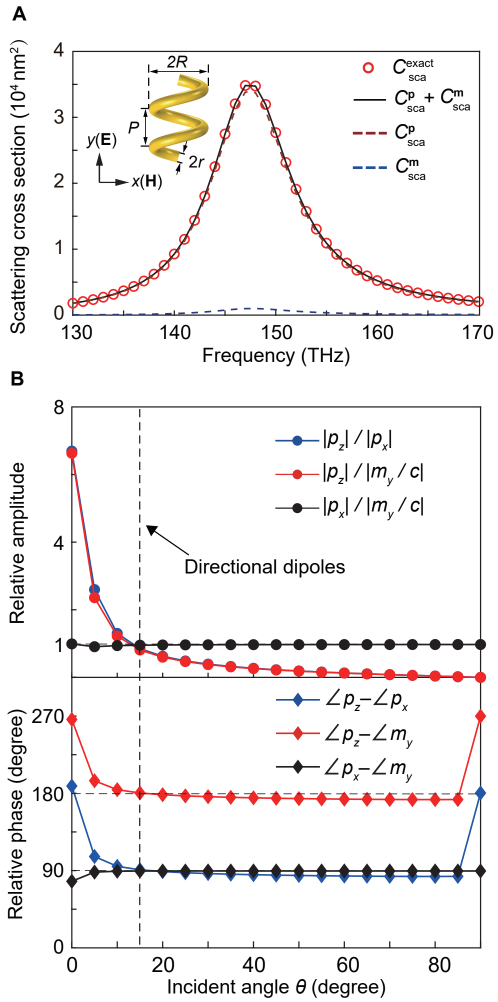

Fig. S2. Electric and magnetic dipole moments induced in the helix. (*A*) Scattering cross sections of the helix particle and the contributions of the electric and magnetic dipoles. (*B*) The relative amplitudes and phases of the dipole components as a function of the incident angle of the linearly polarized plane wave. The dashed line marks the parameters giving three directional dipoles.

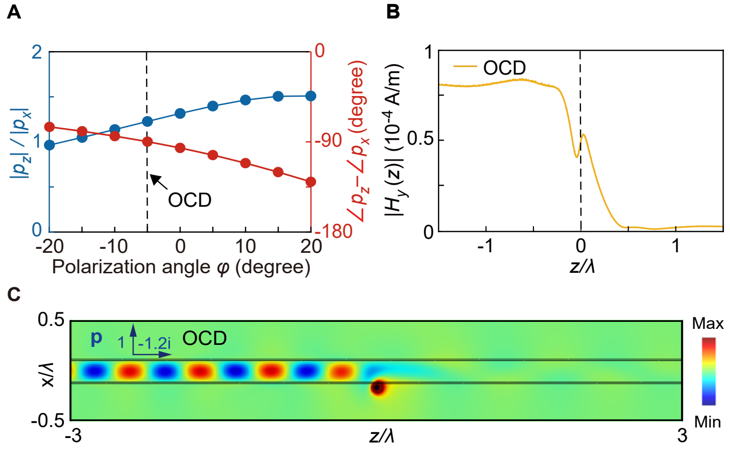

Fig. S3. Reversed directionality of circular-dipole face. (*A*) The relative amplitude and phase of the dipoles p_z and p_x as a function of polarization angle φ. The incident angle is $\theta = 165$ degrees. (*B*) Magnetic field amplitude in the waveguide for the optimized circular dipole (OCD) and (*C*) the unidirectional excitation of the guided wave propagating in $-z$ direction.

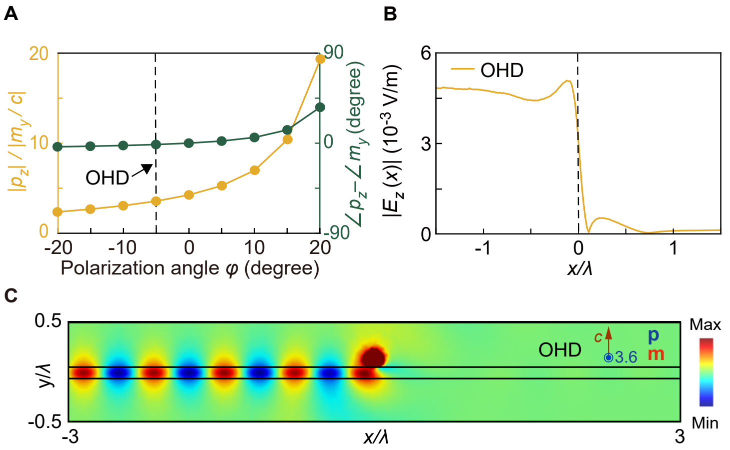

Fig. S4. Reversed directionality of Huygens-dipole face. (*A*) The relative amplitude and phase of the dipoles p_z and m_y as a function of polarization angle φ. The incident angle is $\theta = 175$ degrees. (*B*) Electric field amplitude in the waveguide for the optimized Huygens dipole (OHD) and (*C*) the unidirectional excitation of the guided wave propagating in $-x$ direction.

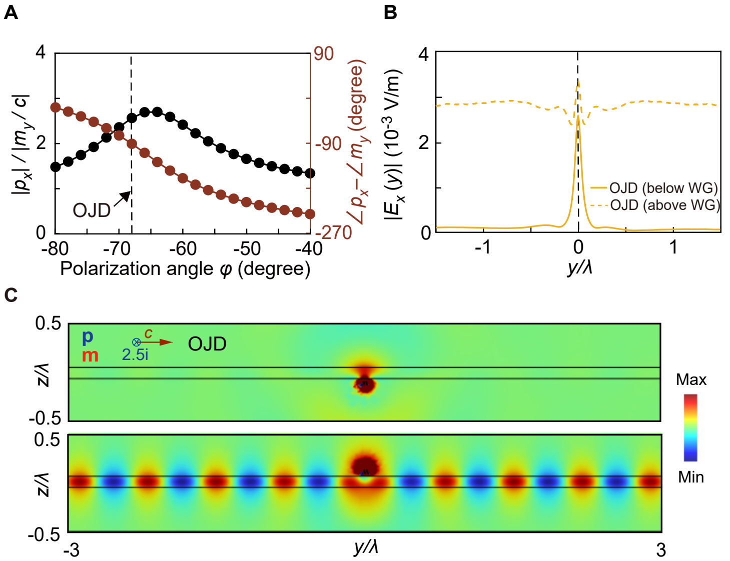

Fig. S5. Reversed directionality of Janus-dipole face. (*A*) The relative amplitude and phase of the dipoles p_x and m_y as a function of polarization angle φ. The incident angle is $\theta = -28$ degrees. (*B*) Electric field amplitude in the waveguide (WG) when the OJD is located below (solid line) and above (dashed line) the waveguide. (*C*) Unidirectional coupling of the OJD.

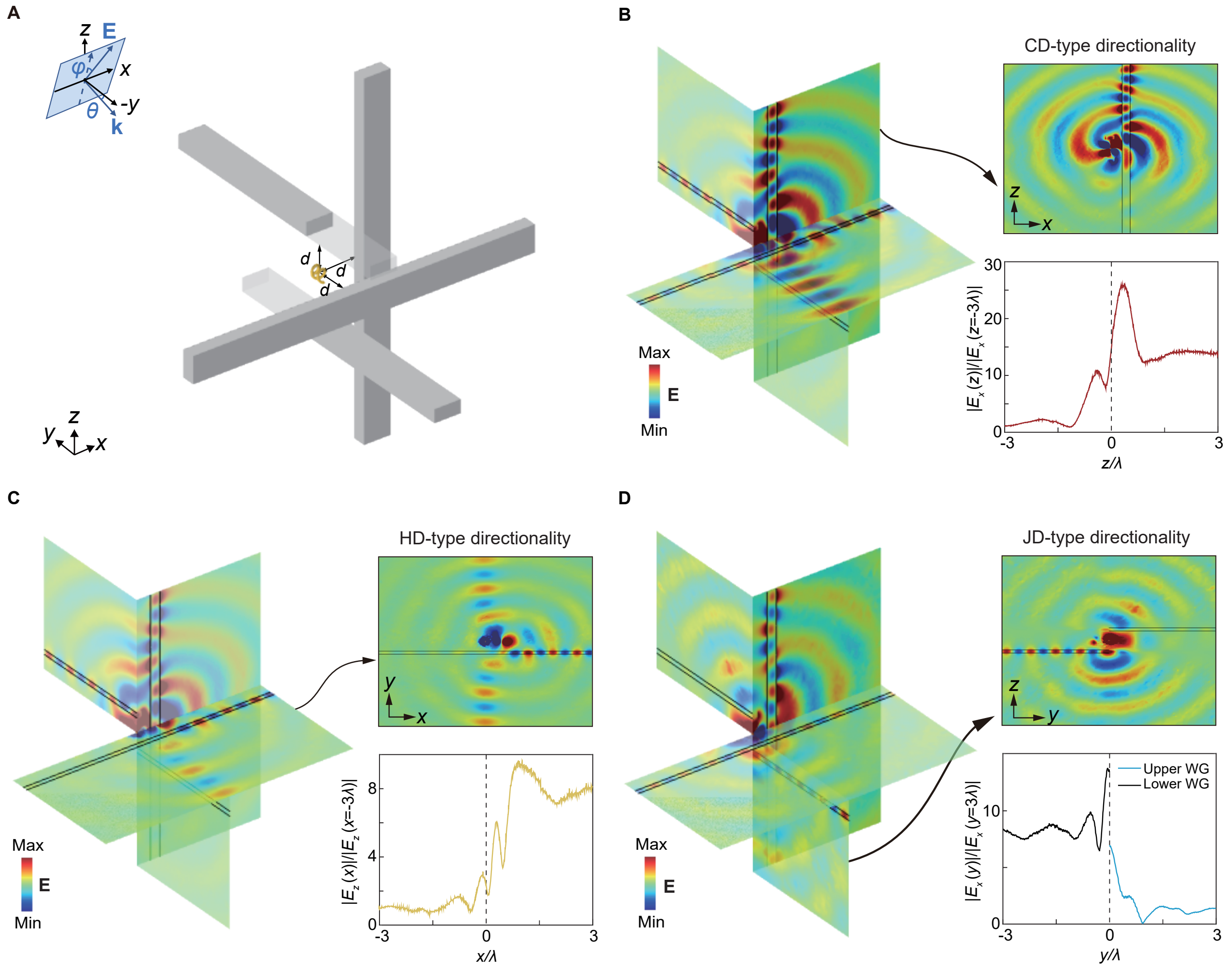

Fig. S6. High-dimensional and multifunctional control of directionality. (*A*) Schematic of the high-dimensional directional system realized by the DDD. (*B*) Electric field distribution of the system at $\theta = 8$ degrees and $\varphi = -5$ degrees for the circular dipole (CD)-type directionality in $+z$ direction. The inset shows the optimized unidirectional coupling on the zx- plane as well as the directionality. (*C*) Electric field distribution of the system at $\theta = 20$ degrees and $\varphi = -25$ degrees for the Huygens dipole (HD)-type directionality in $+x$ direction. The inset shows the optimized unidirectional coupling on the xy- plane as well as the directionality. (*D*) Electric field distribution of the system at $\theta = -36$ degrees and $\varphi = -67$ degrees for the Janus dipole (JD)-type directionality in $-y$ direction. The inset shows the optimized unidirectional coupling on the yz- plane as well as the directionality (WG – waveguide).

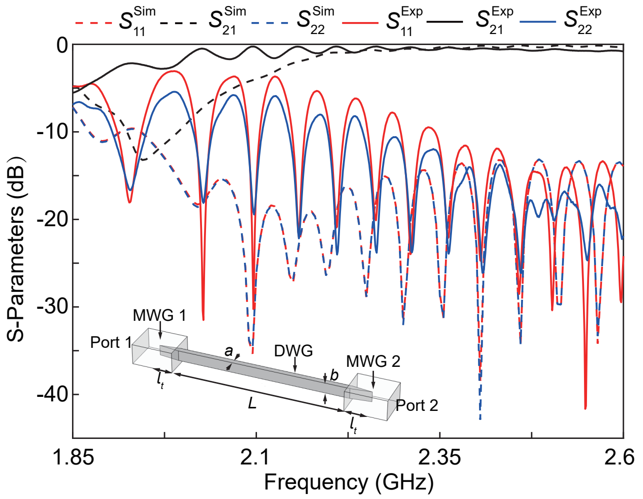

Fig. S7. Dielectric waveguide to metallic waveguide transition. The inset shows the simulation and experiment model (DWG – Dielectric waveguide with dimensions $a = 15$ mm, $b = 30$ mm, $l_t = 60$ mm, $L = 500$ mm, MWG – Metallic waveguide). Extracted simulation and experiment results show minimal reflection and excellent transmission from the input port (Port 1) to the output port (Port 2).

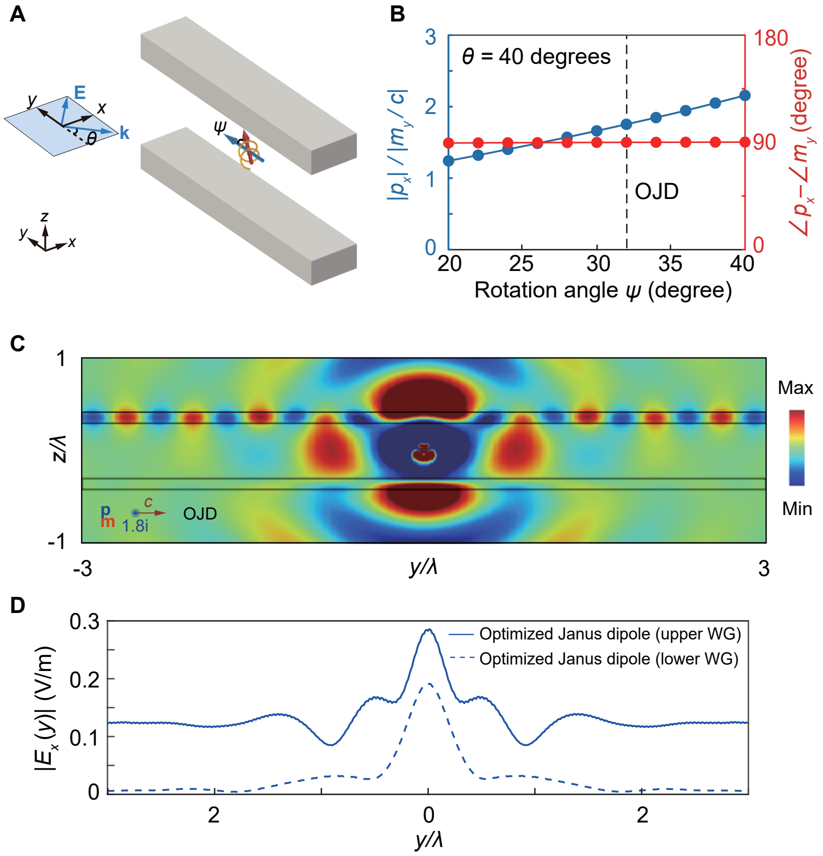

Fig. S8. Directional excitation of guided wave by the Janus dipole in the rotated helix. (*A*) Schematic of the helix-dual-waveguide coupling configuration for demonstrating the directionality of the rotated Janus dipole. The blue and red arrows denote the y direction and the center axis direction of the helix, respectively. (*B*) The relative amplitude and phase of the dipoles p_x and m_y as a function of the rotation angle ψ. The incident angle θ is set to be 40 degrees. (*C*) The optimized Janus dipole of the helix predominantly couples light to the upper waveguide. (*D*) The electric field $|E_x|$ in the upper and lower waveguides (WG).

References

1. T Trinh, J Malherbe, R Mittra, A metal-to-dielectric waveguide transition with application to millimeter-wave integrated circuits in *1980 IEEE MTT-S International Microwave Symposium Digest.* (IEEE), pp. 205–207 (1980).
2. S Dudorov, Ph.D. thesis (Helsinki University of Technology) (2002).
